# Large-Scale Genomic Epidemiology of Klebsiella pneumoniae Identified Clone Divergence with Hypervirulent Plus Antimicrobial-Resistant Characteristics Causing Within-Ward Strain Transmissions

**DOI:** 10.1128/spectrum.02698-21

**Published:** 2022-04-13

**Authors:** Na Pei, Yanming Li, Chunjiao Liu, Zijuan Jian, Tianzhu Liang, Yiming Zhong, Wanying Sun, Jingxuan He, Xinyi Cheng, Hongling Li, Xiaole Lei, Xin Liu, Ziqing Deng, Qingxia Liu, Xia Chen, Qun Yan, Karsten Kristiansen, Junhua Li, Wenen Liu

**Affiliations:** a BGI-Shenzhen, Shenzhen, Guangdong, China; b Department of Clinical Laboratory, Xiangya Hospital, Central South University, Changsha, Hunan, China; c Laboratory of Genomics and Molecular Biomedicine, Department of Biology, University of Copenhagen, Copenhagen, Denmark; d Shenzhen Key Laboratory of Unknown Pathogen Identification, Shenzhen, Guangdong, China; e National Clinical Research Center for Geriatric Disorders, Xiangya Hospital, Changsha, Hunan, China; Peking University People's Hospital

**Keywords:** clone divergence, genomic epidemiology, *Klebsiella pneumoniae*, nosocomial transmission

## Abstract

Global dissemination of K. pneumoniae clones poses health hazards to the public. Genomic epidemiology studies with comprehensive data set further revealed clone divergence, showing a high complexity in evolution. Moreover, clones carrying both acquired virulent and antimicrobial-resistant genes emerged and might replace the carbapenem-resistant clones. Co-occurrence of virulence and resistance is emerging. An unbiased collection of 3,061 clinical K. pneumoniae isolates (January 5, 2013 to July 24, 2018) underwent whole-genome sequencing. Pairwise core-genome single-nucleotide polymorphism (cgSNP) distances identified clone divergence and transmission events. A sum of 2,193 nonduplicated genomes clustered into four phenotypically indistinguishable species complexes. 93% (*n *= 2,035) were KpI with its largest clonal group (CG) being CG11 (*n *= 406). Three hundred ninety-three were ST11 and three hundred seventy-four carried *bla*_KPC-2_. Noticeably, CG11 is divided into two main subclones based on the capsule synthesis K loci (KL). CG11-KL64 showed a clear hypervirulent plus antimicrobial-resistant (hv+AMR) characteristic. Besides, the phylogenetic structure revealed the clone divergence of CG25, and this is the first report with sufficient CG25 genomes to identify the divergence. The outcomes of the hv+AMR CG25 cluster 1 affected patients were poorer (*P* < 0.05). Moreover, two episodes of strain transmissions were associated with CG25 cluster 1. Other transmissions were associated with ST20 and ST307. Genomic epidemiology identified clone divergence of CG11 and CG25. The hv+AMR subclones pose greater threats on a global scale. Nosocomial transmissions of the high-risk clones raised our concerns about the evolution and transmission of emerging clones among newborns and critically ill patients.

**IMPORTANCE** The convergence of AMR and acquired virulence posing higher risks to the public is a focusing point. With sufficient genomes and genotypes, we successfully identify the convergence in two subclones, the previously reported CG11-KL64, and the newly reported CG25 cluster 1. The novel finding of the CG25 divergence was not only revealed by the phylogenetic tree but also confirmed by the clinical outcome data and the accessory genome patterns. Moreover, the transmission subclones circulated in two clinically important wards highlights the deficiency of infection control program using conventional methods. Without the assistance of whole-genome sequencing, the transmissions of high-risk clones could not be identified.

## INTRODUCTION

The control of Klebsiella pneumoniae in health care settings is of great public health concern due to the globally spread hypervirulent (hv, acquired ≥ four virulence factors) sequence types of ST23, ST65, ST86 clones, and multidrug-resistant (MDR, acquired resistance to at least one agent in ≥ three antimicrobial categories) ST11 (ST258), ST15, ST147 clones (1, 2). The hv clones that can acquire additional virulent associated factors are more virulent than the classic K. pneumoniae clones, and seldomly carried antimicrobial-resistant genes (ARGs) (3). The population structures are often distinguishable by both genomic features and pathogenetic characteristics. However, reports on clone divergence of ST11 and ST15 complexed the issue, genomes from the same clone can exhibit different phenotypical features and introduce diversified clinical outcomes (2, 4). The divergent events of ST11 and ST15 were mainly due to the chromosomal recombination of the capsule polysaccharide K loci (KL), and the acquisition of virulent, or hv+AMR plasmids (4–6). The ST11-KL64 and ST15-KL2 clone shifts were distinguishable by their enhanced virulence with significantly poorer outcomes and higher abilities to cause nosocomial outbreaks (2, 7).

In addition to the novel subclone emergence, the emergence, and rise of the high-risk clones, such as ST20, ST25, ST37, ST101, ST307 have also been reported, albeit sporadically (8, 9). The emerging high-risk clones are potential threats to public health as they can evolve into dominant clones by the same mechanism of horizontal gene transfer (HGT) and cause local outbreaks (2). For instance, one of the high-risk clones, ST20, has caused small outbreaks in the neonate wards in Wuhan and Shandong, China by the acquisition of *bla*_NDM-1_ (10, 11). Similarly, ST25 carrying *bla*_NDM-1_ and *bla*_KPC-2_ has been reported to be associated with its clone expansion on a global scale (8, 12). Likewise, it is reported that ST307 is replacing the hyperendemic clone of ST11 (ST258) by obtaining carbapenem-resistant (CR) genes of *bla*_KPC_, *bla*_NDM_ and *bla*_OXA-48_-like in the Americas, South Europe, and South Africa (1, 8, 9, 13, 14).

To investigate clone divergence and clinical status of the emerging clones, we carried out this whole-genome sequencing (WGS) analysis study and used the ultrahigh-resolution genomic data to distinguish genomes of the same clone and correlate the genomic data with the epidemiological data as well as the patient outcomes. Here, we provided a detailed epidemiological overview of 2,193 K. pneumoniae genomes of a 5-year collection and determined the clone divergence events at both local and global levels. Finally, we identified within-ward transmission events caused by the emerging clones which were neglected by the conventional surveillance program.

## RESULTS

### Genomic epidemiology, acquired virulence, and antimicrobial susceptibilities.

**FIG 1:**
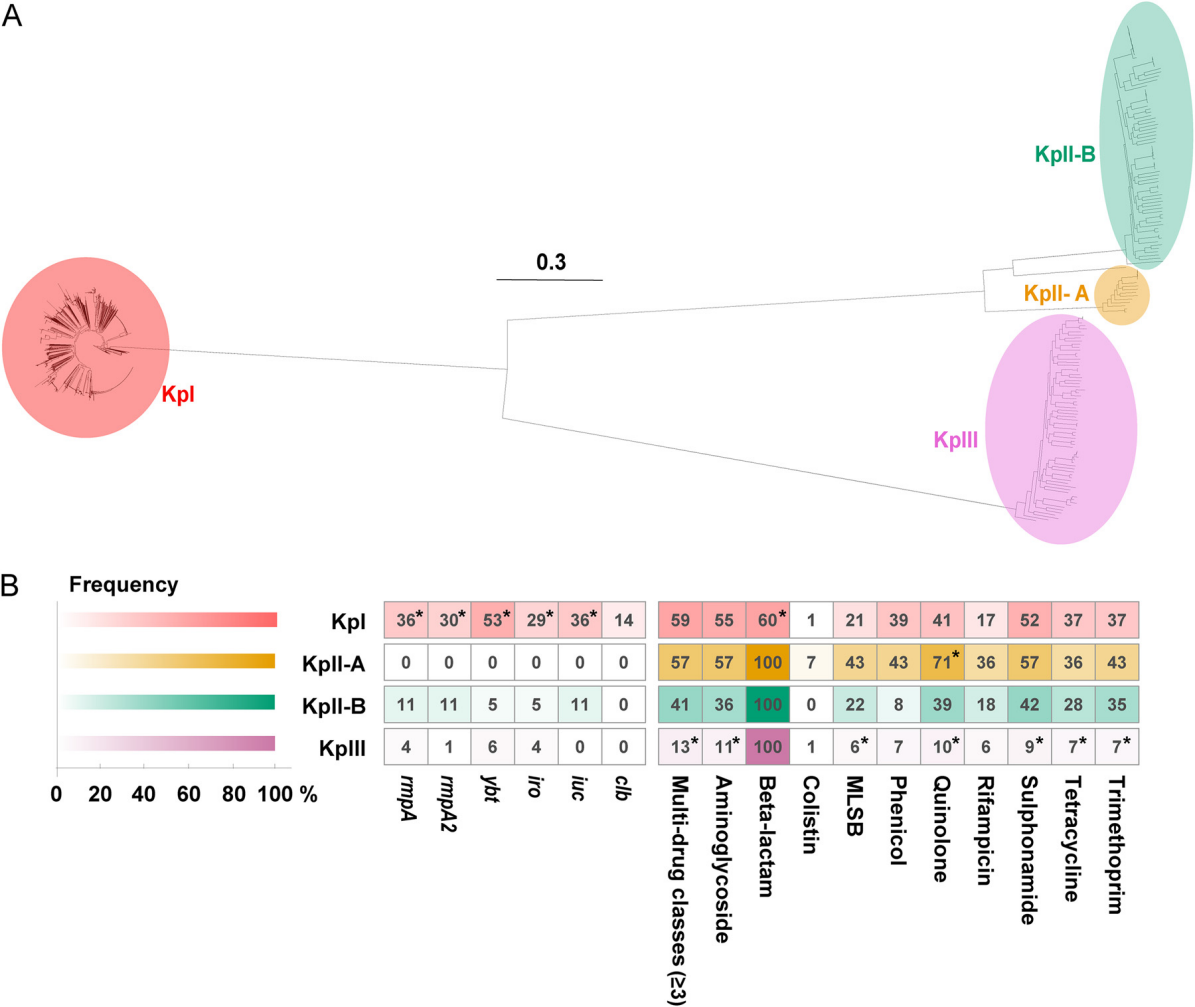
Distribution of the four Klebsiella pneumoniae species complexes (*n *= 2,193). (A) Maximum likelihood tree constructed by the core genome single-nucleotide polymorphisms. The scale bar indicates the nucleotide substitutions per site. (B) Heatmap showing the profiles of the acquired virulent genes and antimicrobial-resistant genes. The virulent genes were the capsule production regulator genes of *rmpA* and *rmpA2*, the siderophore encoding genes of *ybt*, *iro*, and *iuc*, and the colibactin gene, *clb*. The drug classes to which the genes conferring resistance were listed (the intrinsic genes of *bla*_SHV_, *fosA*, *oqxA*, and *oqxB* were excluded). *, adjusted *P* < 0.05 by the Fisher's exact test. KpI, K. pneumoniae. KpII-A, K. quasipneumoniae subsp. quasipneumoniae. KpII-B, K. quasipneumoniae subsp. similipneumoniae. KpIII, K. variicola. MLSB, macrolide-lincosamide-streptogramin B.

Genomes of 2,193 unique K. pneumoniae isolates were collected from 2,193 patients (Table S5). The involved individuals were mainly from the intensive care unit (ICU, *n *= 582 [27%]). Only 125 (6%) were neonates. Maximum likelihood (ML) phylogenetic analysis grouped the 2,193 genomes into four phenotypically indistinguishable species complexes, KpI (K. pneumoniae, *n *= 2,035 [93%]), KpII-A (K. quasipneumoniae subsp. quasipneumoniae, *n *= 14 [1%]), KpII-B (K. quasipneumoniae subsp. similipneumoniae, *n *= 74 [3%]), and KpIII (K. variicola, *n *= 70 [3%]) (Fig. 1A). Principal-component analysis (PCA) based on the 5,650 accessory genes also distinguished the 2,193 genomes into four groups with only a small number of shared genes (Fig. S2). The majority contained four intrinsic ARGs (*bla*_SHV_, *fosA*, *oqxA*, and *oqxB* conferring very low-level resistances to the beta-lactam, fosfomycin, and fluoroquinolone antibiotics) on chromosomes (Fig. S3). In terms of the carriage of acquired VFs and ARGs (excluding the intrinsic ARGs), significant differences existed in KpI, KpII-A, and KpIII among the other species complexes (adjusted *P* < 0.05) (Fig. 1B). KpI group implied an apparent trend of co-occurrence of acquired virulence and AMR. Five of six acquired virulent genes were significantly higher than the other three groups (adjusted *P* < 0.05).

**FIG 2:**
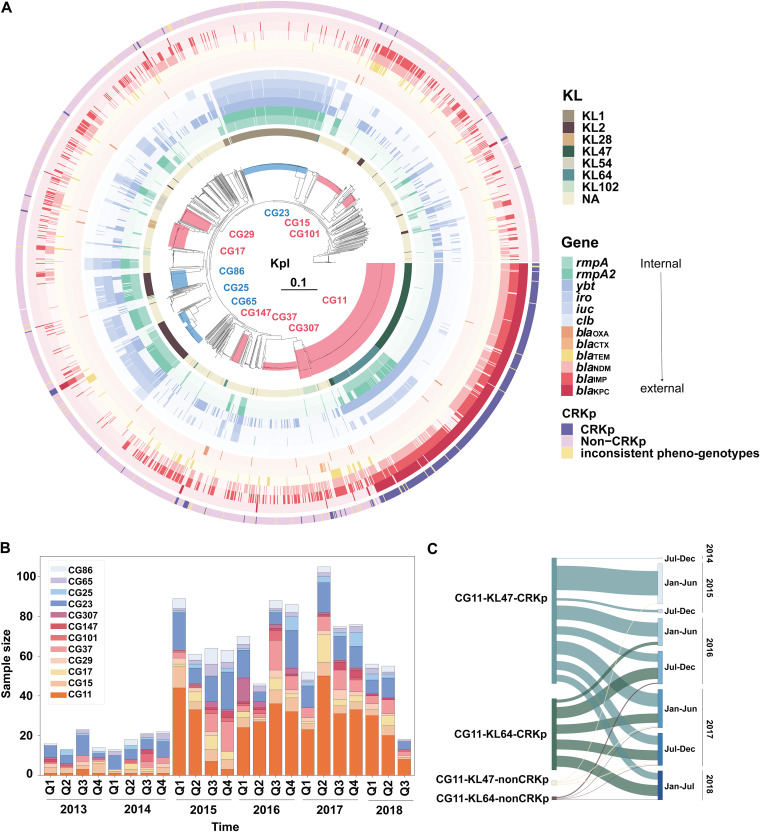
Clonal group distributions of the Klebsiella pneumoniae (KpI) genomes (*n *= 2,035). (A) Maximum likelihood phylogenetic tree constructed by 254,411 core genome single-nucleotide polymorphisms. The scale bar represents the number of nucleotide substitutions per site. The multidrug-resistant (MDR) and hypervirulent (hv) clonal groups (CGs) containing more than 30 genomes are shaded and labeled in red and blue, respectively. The K loci (KL) and the presence of virulent and β-lactamase *bla* genes are shown by three sets of rings. The outmost ring shows the carbapenem-resistant K. pneumoniae (CRKp), non-CRKp, and isolates with inconsistent carbapenem-resistant phenotype and genotype. (B) Seasonal distribution of the main clonal groups. Q, quarter. (C) Sankey diagram representing the distribution of CRKp of the two main KL types of CG11 in half-year intervals.

Multilocus sequence typing (MLST) gave 394 sequence types (STs) to the 2,035 KpI genomes, including 315 reported ones and 79 novel types assigned to 91 genomes (Table S6). Core-genome SNPs grouped the 2,035 KpI genomes into 30 clonal groups (CGs) with the largest being CG11 (*n *= 406 [20%], containing ST11, ST258, ST512, and derivatives) followed by CG23 (*n *= 216 [11%], containing ST23 and derivatives), CG37 (*n *= 109 [5%]), CG15 (*n *= 94 [4%]), CG17 (*n *= 72 [3%]), CG65 (*n *= 62 [3%]), and CG86 (*n *= 57 [3%]) (Fig. 2A). CG11 was the largest, and contained an average of 12 ARGs, with *bla*_KPC-2_ (374 [92%] of 406) being the most predominant, and, thus, a high proportion of phenotypically carbapenem-resistant K. pneumoniae (CRKp, 367 [90%] of 406) was uniquely observed in CG11 (*P* < 0.001) (Table S7). The main carbapenemase genes in CRKp were *bla*_KPC_ (397 [84%] of 472) followed by *bla*_NDM_ (30 [6%] of 472) and *bla*_IMP_ (12 [3%] of 472). The extended-spectrum beta-lactamase (ESBL) gene *bla*_CTX-M-65_ was significantly higher in CRKp isolates (292 [93%] of 314, *P* < 0.001); while the other *bla*_CTX-M_ alleles nor ESBL genes were not.

Markedly, the carbapenem-resistant CG11 clone was divided into two main subclones based on the capsule synthesis K loci (KL) (Fig. 2A). CG11-KL64 carried more virulent genes (mainly *rmpA*/*A2*, *ybt*, and *iuc*) than CG11-KL47 (*P* < 0.001), showing a hv+AMR characteristic. The CG11 blowout began in 2015 and persisted until 2018 (Fig. 2B). CG11-KL47 emerged earlier than CG11-KL64, CG11-KL64 appeared until the end of 2016 (Fig. 2C). Both CG11-KL47 and CG11-KL64 contained many CRKp isolates. Within-quarter sudden-uprisings, such as CG307 (all ST307) in the first quarter of 2016, CG17 (including ST17, ST20, and the derivatives) in the second quarter of 2017, and CG25 (all ST25 except for one ST304) in the last quarter of 2016 and 2017 were also observed (Fig. 2B). In contrast, CG23 isolates were continuously identified throughout the study period with no substantial changes.

### CG25 clone divergence.

**FIG 3:**
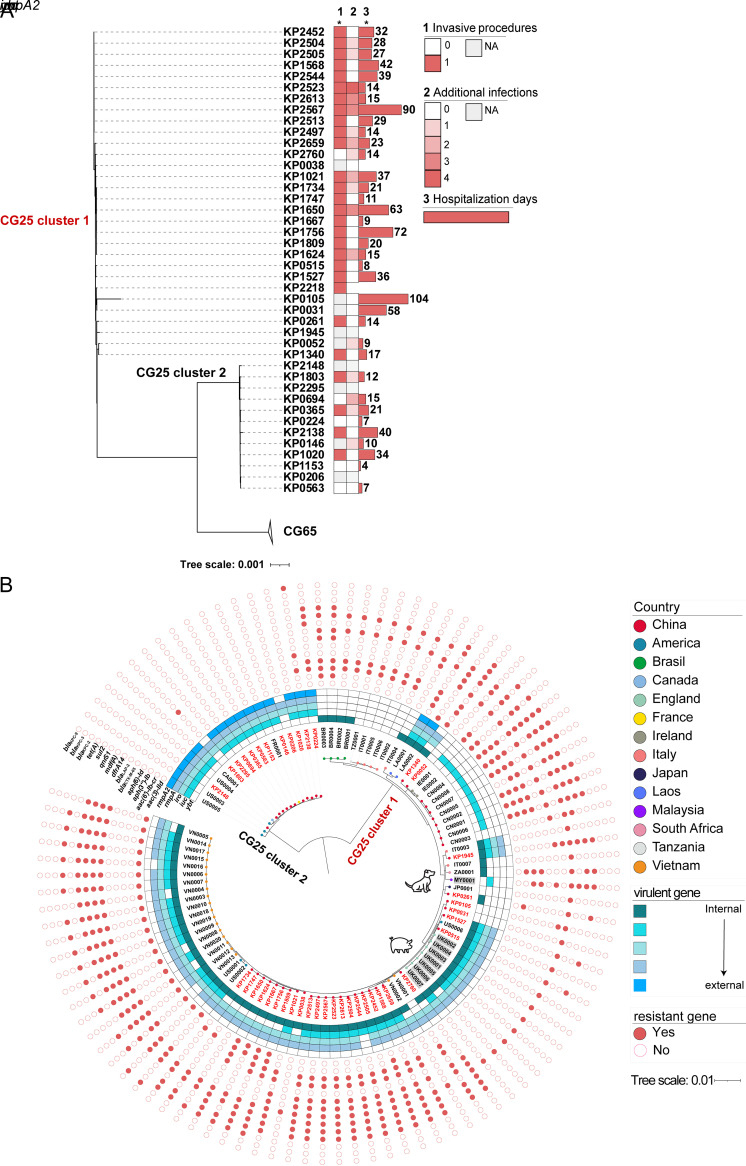
Maximum likelihood trees showing the clone divergence of clonal group 25 (CG25, including ST25). (A) A local tree was constructed by 42 CG25 and 62 CG65 genomes of this study (*n *= 104). The clinical histories are shown. The CG65 cluster is collapsed, and the two CG25 clusters were clearly labeled. The tree scale represents the number of nucleotide substitutions per site. *, *P* < 0.05. 1, invasive procedures. 2, additional infections acquired during hospitalization (Table S8). 3, hospitalization days (day counts are indicated). 0, absent. 1, present. NA, not available. (B) A global tree constructed by ST25 genomes using 3,532 core genome single-nucleotide polymorphisms (*n *= 103). Tip node colors show the countries where the strains were isolated. Labels of genomes sequenced by this study are bold in red, the public genomes were in black (Table S4). Those isolated from animals are shaded in gray. The presence of virulent genes and antimicrobial-resistant genes statistically different between clusters 1 and 2 are shown by cold and warm colors, respectively (*P* < 0.05).

Remarkably, CG25 of this study was divided into two subclones by the phylogenetic structure (Fig. 3A). A total of 25,549 cgSNPs (16,728 synonymous and 5,567 missense) characterized the two subclones, with no predominant recombination events identified. CG25 cluster 1 included 29 ST25 and one ST304 genome, whereas CG25 cluster 2 only contained 12 ST25 genomes. A closer relatedness was identified between CG25 cluster 2 and CG65, CG25 cluster 2 was like a derived clade of CG65. Patients affected by CG25 cluster 1 had poorer outcomes compared with patients affected by CG25 cluster 2 (Table S8). Specifically, 23 (96%) of the 25 CG25 clusters 1 patient with clear treatment records had experienced invasive treatment; however, only four of the eight (50%) patients infected by CG25 cluster 2 underwent invasive procedures (*P* < 0.05). Moreover, CG25 cluster 1 patients had more than two additional infections during their stay in this hospital, along with one dead (P1393) and four transfer cases in life-threatening conditions (P0083, P1943, P1962, and P2028), whereas CG25 cluster 2 affected patients only had one and two additional infections during hospitalization. Furthermore, the hospitalization days between the two clusters were significantly different (*P* < 0.05).

The divergence of CG25 in branch distances and clinical outcomes made us wonder if this was a local case or a global one. So, we additionally analyzed 62 public ST25 draft genomes, which were isolated from 14 countries across Asia, America, Africa, and Europe to determine the relationships between the dispersed CG25 cluster 1 and 2 (Table S4). After mapping the accessory genomes to the spilt global ST25 tree, we confirmed the ST25 clone divergence was not limited to China. Most ARGs concentrated in CG25 cluster 1 along with acquired virulence factors, showing a hv+AMR characteristic, while few ARGs exited in CG25 cluster 2 (Fig. 3B). The carriage of ARGs resistant to fluoroquinolone and aminoglycoside, beta-lactam, trimethoprim-sulfamethoxazole were common in the hv+AMR CG25 cluster 1, indicating a high-risk clone. Moreover, we noticed the hv+AMR CG25 cluster 1 isolates could be obtained from both humans and animals (a dog from Italy and seven pigs from the United Kingdom), while the CG25 cluster 2 only contained human isolates. The hv+AMR CG25 cluster 1 had a larger geographic distribution with the initial strain UI 12780 (LA0001 in Fig. 3B, SRA accession number ERR025590) isolated in 2008 containing six ARGs. In contrast, the first published ST25 genome in CG25 cluster 2, CAS689 (CA0001 in Fig. 3B, ERR706869) was devoid of ARGs.

### Limited genetic diversity within the high-risk transmission subclones.

**FIG 4:**
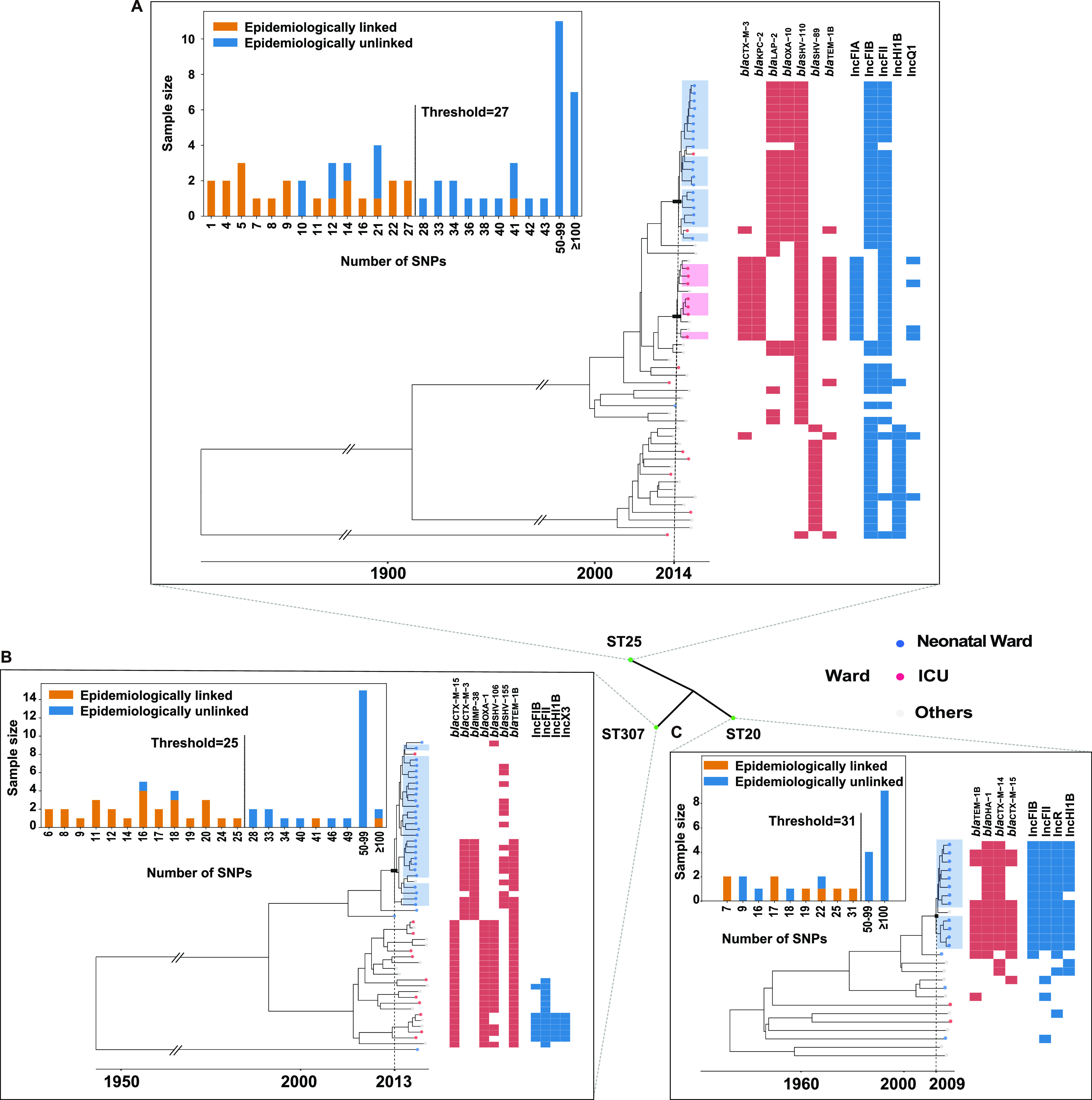
Maximum clade credibility tree and genetic characteristics of the high-risk transmission clones. Distributions of the pairwise core genome single-nucleotide polymorphism are displayed by bar charts. Tree tip colors show the ward types, and the epidemiologically linked tips are shaded. Dashed lines connect the most common recent ancestor to the date estimated for its emergence. The carriage of β-lactamase *bla* genes and plasmid replicons statistically different between the subclones is represented by the red and blue boxes (*P* < 0.05). (A) ST25. (B) ST307. (C) ST20.

To understand the contribution of strain transmission within each clone, especially the hv, AMR, and hv+AMR subclones, a closer look was taken at the pairwise cgSNP level. At the very beginning, a rough scanning of ward distribution of genomes linked by two cgSNPs and a half-year interval was initially carried out. The clustering result indicated possible strain transmission would have happened heavily between ICU and surgical wards, and in a neonatal ward (Fig. S4). To investigate the potential transmission events more accurately, the F1 scores of the pairwise cgSNP values separating each clone were calculated (Table 1, Fig. S5). The ML tree of each clone was reviewed for clustering. Genomes clustered in the largest subclone taking up more than 30% of the total genomes of the subclone (F1 > 0.75) were selected for transmission investigation. The cgSNP counts (27 for ST25, 25 for ST307, and 31 for ST20) well spilled the epidemiologically linked with the unlinked (Fig. 4). Most of the unlinked were from various clinical departments over a long interval. The dated phylogenetic tree revealed the epidemiologically linked genomes were also genomically linked. Four transmission subclones of ST25-icu, ST25-neo, ST307-neo, and ST20-neo were formed. The times of the most recent common ancestor (tMRCA) for those subclones were estimated to be December 2014 for ST25-icu (95% highest posterior density [HPD] interval; June 22, 2014 to August 7, 2015), May 2015 for ST25-neo (95% HPD interval; October 11, 2014 to December 3, 2015), February 2013 for ST307-neo (95% HPD interval; August 9, 2011 to March 28, 2014), and December 2009 for ST20-neo (95% HPD interval; June 2, 2001 to June 2, 2015). All transmission subclones represented recently emerged rather than historical genotypes. Moreover, the accessory genomes showed discriminations between the transmission subclones and the unlinked remainders. The *bla*_CTX-M-3_, *bla*_KPC-2_, and *bla*_TEM-1B_ genes along with the IncFIA and IncQ1 replicons were uniquely identified in ST25-icu (Fig. 4A). In contrast, *bla*_LAP-2_, and *bla*_OXA-10_ were more common in ST25-neo. Both ST25-icu and ST25-neo were associated with the hv+AMR CG25 cluster 1. The accessory genomes of ST307-neo divided the transmission subclone into two parts, one was relatively plain, and one was related to *bla*_CTX-M-3_, *bla*_IMP-38_, and *bla*_TEM-1B_ (Fig. 4B). It might indicate the resistance-acquisition subclone was derived from the ancestral antimicrobial-susceptible subclone. Again, in the same neonatal ward, ST20-neo was developed with the carriage of multiple ARGs (*bla*_TEM-1B_, *bla*_DHA-1_, *bla*_CTX-M-14_, *bla*_CTX-M-15_), and plasmid replicons of IncFIB, IncFII, IncR, and IncHI1B, showing an apparent competency of horizontal gene transfer (HGT) than its distantly related counterparts (Fig. 4C). The transmission associated isolates were highly correlated with MDR infections (89% to 100%), and almost all associated patients had received the ‘last resort’ antibiotic treatments (such as carbapenem, 40% to 89%), and invasive treatments (89% to 100%) (Table 2).

**TABLE 1:** Sequence type subclones defined by the pairwise core genome single-nucleotide polymorphism thresholds

Sequence type clones	No. of unique isolates (>20)	No. of genomes	Mean pairwise cgSNP (SD)	Pairwise cgSNP threshold	No. of Sub-clones containing epidemiologically linked genomes	Genomes in the largest subclone (%)	F1 score
ST25	41	60	399 (384)	27	2	18 (30%)	0.82
ST307	31	54	168 (141)	25	1	26 (48%)	0.93
ST20	22	26	343 (242)	31	1	12 (46%)	0.76
							
ST11	393	674	26 (20)	0	52	10 (1%)	0.35
ST15	62	97	325 (1, 510)	11	4	5 (5%)	0.47
ST17	33	36	258 (88)	6	1	2 (6%)	0.00
ST23	203	235	404 (2, 070)	2	1	2 (1%)	0.67
ST35	29	33	354 (91)	3	1	2 (6%)	0.67
ST36	21	27	735 (286)	14	1	2 (7%)	1.00
ST37	76	90	290 (415)	6	3	4 (5%)	0.32
ST45	25	30	301 (566)	17	2	4 (13%)	0.67
ST592	22	24	1,487 (3, 668)	39	1	2 (8%)	0.50

**TABLE 2:** Summary of clinical records of the patients involved in the within-ward transmission events

Clinical characteristics	ST25-icu	ST25-neo	ST307-neo	ST20-neo
Unique patients (male)	5 (5 [100%])	9 (5 [56%])	19 (10 [53%])	10 (7 [70%])
MDR^*a*^ strain carriage (%)	5 (100%)	8 (89%)	19 (100%)	10 (100%)
'Last resort' treatments (%)				
Carbapenem	2 (40%)	8 (89%)	13 (68%)	7 (70%)
Invasive procedures (%)	5 (100%)	9 (100%)	17 (89%)	9 (90%)
Intubation, ECMO^*b*^	3	7	0	0
Nasogastric tube	5	6	13	4
Decompression tube	1	9	7	5
Urinary catheter	5	0	2	0
Vessel catheter	4	9	6	9
Others	0	1	3	0
Infection classification				
RTI^*c*^	5	7	7	8
Sepsis	0	7	13	5
GI^*d*^	2	4	4	1
UTI^*e*^	2	2	0	0
CNSI^*f*^	1	0	5	0
Others	2^*g*^	2^*h*^	0	0
Hospitalization duration				
Average	2.5 wks	4.6 wks	5.8 wks	7.1 wks
Range	9-33 days (one dead case)	14-90 days (four transfer cases)	8-84 days	3-87 days (one transfer case)

a MDR, multiple drug resistance.

b ECMO, extracorporeal membrane oxygenation.

c RTI, respiratory tract infection.

d GI, gastrointestinal infection.

e UTI, urinary tract infection.

f CNSI, central nervous system infection.

g Include myositis ossificans and exposure keratitis.

h Includes two wound infections.

**FIG 5:**
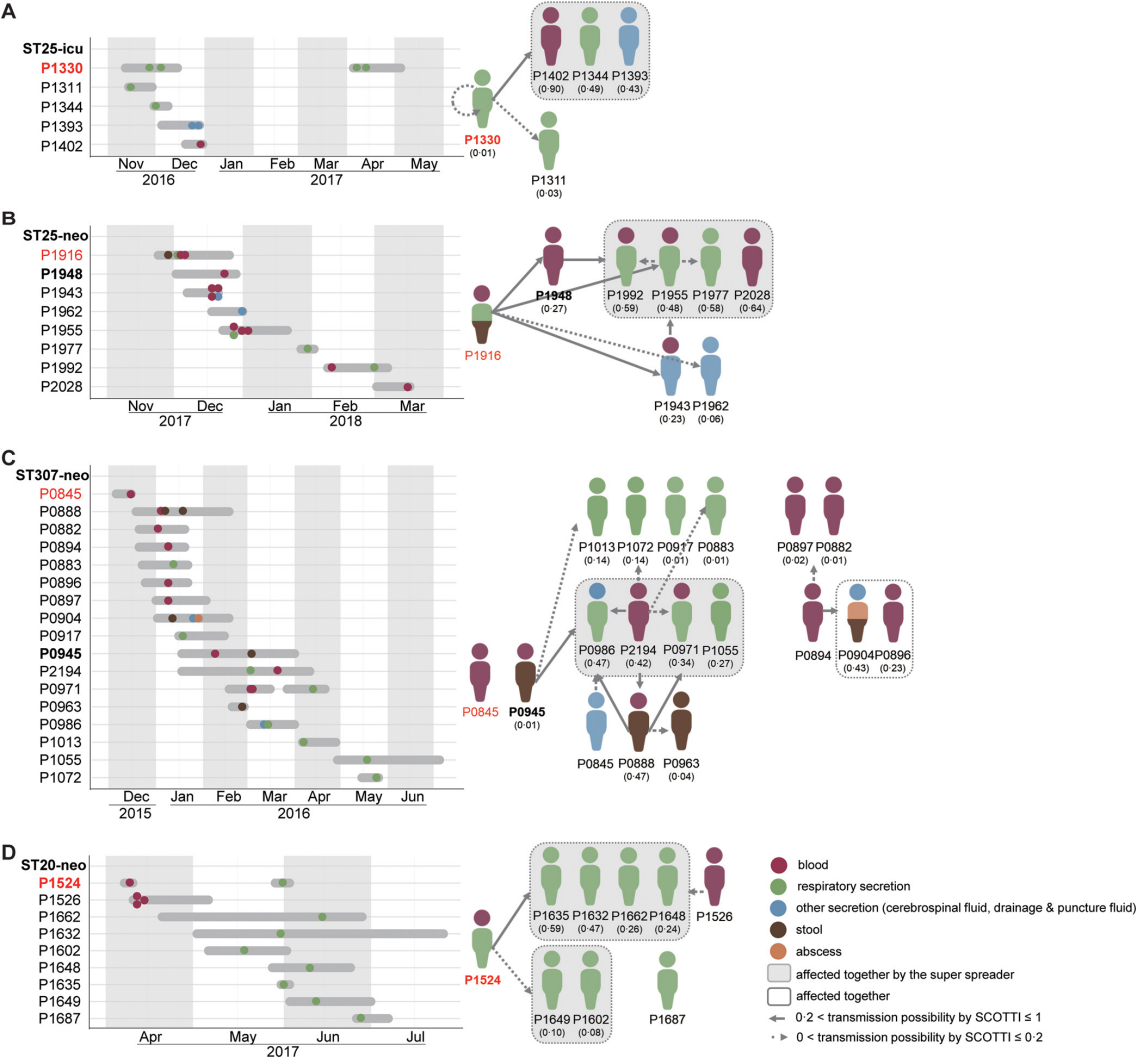
Timelines and transmission chains constructed for the high-risk transmission sequence type subclones. In the timelines, the *y*-axis represents patient codes; red indicates the index (first) cases, and the bold texts highlight the super spreaders. The gray horizontal lines indicate the hospitalization periods, and the dots of different colors show the sampling time points and the specimen types. The specimen color codes also apply to the transmission networks. The predicted possibilities of the transmissions are shown in the brackets. (A) ST25-icu, (B) ST25-neo, (C) ST307-neo, and (D) ST20-neo.

To reconstruct the transmission routes and infer the transmission directions, SCOTTI analysis was carried out with the date data of hospitalization and sampling along with the genomic sequences. The analysis showed that ST25-icu happened in late 2016 with a predicted root case for P1330 (Fig. 5A). The patient P1330 was a 47-year-old male, who had a cerebral hemorrhage. Although discharged from ICU in the December of 2016, he was readmitted to this hospital in 2017 with the identification of strains from ST25 cluster 1, again. ST25-neo persisted from 2017 to 2018 (Fig. 5B). The first two cases, P1916 and P1948, formed the keys to the transmission. P1916 was the index case, and P1948 was the superspreader. Both were with very low birth weight infants (VLBWIs) enduring sepsis. The superspreader of ST307-neo was identified as P0945 (male, preemie) (Fig. 5C). P0945 was diagnosed with a *bla*_CTX-M-3_ and *bla*_IMP-38_-carrying isolate. In contrast, the index and earlier case, P0845 with few ARGs, seemed less contagious. This may indicate a more severe threat was given by the later (2016) ST307-neo isolates rather than the earlier (2015) ones. The host of the earliest case of ST20-neo, P1524, was also the superspreader (Fig. 5D). He was a premature infant born in this tertiary hospital in March 2017 and was diagnosed with sepsis (strain KP1939, recovered from blood) in April. In May, he developed pneumonia (strain KP2095 from respiratory secretion). The following cases of ST20-neo were all associated with respiratory infections, P1635 strain KP2100 (probability = 0.59), P1632 strain KP2093 (0.47), P1662 strain KP2140 (0.26), and P1648 strain KP2124 (0.24).

## DISCUSSION

We undertook a retrospective WGS survey of K. pneumoniae covering the years from 2013 to 2018 and identified two clone divergent events and four outbreaks among neonates and ICU patients. This is the first study with enough genomes and genotypes to reveal the clone divergence of CG25 (including ST25). CG25 cluster 1 is an emerging high-risk clone with poorer clinical outcomes and hv+AMR characteristics, while CG25 cluster 2 is a conventional hypervirulent clone. Two episodes of nosocomial transmissions were associated with the hv+AMR CG25 cluster 1, and another two are related to the emerging high-risk clones of ST307 and ST20.

The previously reported K. pneumoniae clone divergent events were restricted to CG11 and CG15 and focused solely on the K loci (2, 5). Insufficient data sets of the early studies of ST25 assigned the isolates to a clonal complex CC65^K2^ (15). Here, we expanded the idea of clone divergence identification to a whole-genome scale with sufficient genomes and identified the CG25 divergence. The divergence is mainly due to mutations and ARG acquisitions. ST25 was originally famous for its KL2 capsular with a hypermucoviscous phenotype (8). Former ST25 strains, CAS689 (CA0001 in Fig. 3B) isolated in 2005, and L3 (FR0001 in Fig. 3B) isolated in 2008 were devoid of ARGs. However, in recent years, reports on the acquisition of carbapenem resistance (via *bla*_KPC_ and *bla*_NDM_) in ST25 began to arise (12). ST25 has, thus, been regarded as an outlier among the hv clones (2). WGS helped identify 11 *bla*_KPC-2_-carrying ST25 isolates in our collection; nine (82%) concentrate in ST25-icu. This finding correlates well with the phenomenon of ST25-icu evolution under the heavy carbapenem treatment for ICU patients (16). Some state the phenomenon as virulence-AMR convergence, and we find the convergence is not only due to the HGT but also due to the chromosomal mutations (17). There were two evolution paths of CG25. The path of the hv+AMR CG25 cluster 1 is superior to the path of the hv CG25 cluster 2. We also correlated the clinical outcomes with the phylogenetic findings. Significantly poor outcomes for CG25 cluster 1 were found. The poor outcome can be explained by the acquired ARGs (18). The global collection of ST25 genomes provides further evidence to support the ST25 divergence; significantly higher AMR-virulence convergent isolates were in the CG25 cluster 1.

The predominance of CG11 (or called CG258, including ST258 and ST11) is mainly based on the acquisition of the carbapenemase gene *bla*_KPC-2_. Simultaneous carriage of ARGs and acquired virulent genes were reported in CG11-KL64 in China and could lead to significantly higher mortalities (4). Our findings were consistent with that trend and specify the spread of CG11 emerged locally in 2015, broke down the domination of CG23, and gradually increased the K. pneumoniae burden in the hospital. Regardless of the continuous evolution of CG11, there were less than 1% of ST11 genomes clustered together in a single ward within 180 days. Thus, we regarded it as a clone expansion with beneficial genes and did not explore it for transmissions in detail. Although CG25, CG307, and CG17 (including ST20) do not show long-time endemic trends similar to CG11, small expansions of those clones were observed (Fig. 2B). So deeper characterization of those clones was carried out in the aspects of genetic distances, resistant and virulent profiles, clinical manifestation, and transmission possibilities.

ST20 and ST307 have been reported as high-risk clones as 40% of the members had the ESBL genes (19). ST20 causing strain transmission in neonatal units has been reported in Greece, China, and Vietnam (11, 20, 21). The transmission subclones were ESBL and carbapenemase (*bla*_NDM_) producing, similar to ST20-neo showing an apparent HGT competency. Moreover, we notice most ST20-neo isolates were from respiratory specimens of preterm infants with pneumonia. Further studies on ST20 transmission among neonatal patients and via respiratory care supplies may be necessary. ST307 has been reported as an emerging MDR clone replacing ST11 (ST258) by the acquisition of *bla*_KPC_, and *bla*_OXA-48_-like in the United States, Southern Africa, Europe, and Germany (13, 14). In China, ST307 has been reported carrying *bla*_CTX-M-15_ in the northern communities, and *bla*_IMP-38_ in the hospital of this study (22, 23). Our results confirm the finding of *bla*_IMP-38_-carrying ST307 in the hospital and specify the high-risk isolates belonging to ST307-neo. K. pneumoniae can cause death in immunocompromised patients (24). The transmission recipients in this hospital, the newborns and the ICU patients, were at increased high risk of low immunities and, thus, should be more carefully protected. In this situation, we advise the implementation of WGS in the surveillance of strain transmission inwards of immunocompromised patients.

However, this study does have limitations. First, because this is a retrospective study, sampling of the related staff, devices, foods, and environmental surfaces was not possible. Thus, it lacks information to define the exact factor contributing to the transmission episodes among people with limited contacts. Because SCOTTI predicted our transmission cases with “unsampled” nodes, indirect transmissions should be common between patients, and the key connecting determinant was unknown. Obtaining sequencing data from other sources may give a clearer picture of the transmission paths.

In conclusion, based on the epidemiological observations of the large K. pneumoniae collection, we identified clone divergence of CG25 and strain transmissions in high-risk clones. The hv+AMR CG25 cluster 1 leading to poor clinical outcomes shows a global trend of clone expansion. A total of four transmission events were identified by our integrated approaches. Two were associated with the hv+AMR CG25 cluster 1, and another two were associated with the high-risk clones of ST20 and ST307. All were emerging clones with great threats to the public. The findings also emphasize the importance of implementing genomic pathogen surveillance in the infection control program, particularly for neonates and severely ill patients.

## MATERIALS AND METHODS

### Study design and sample collection.

This study included 3,061 Klebsiella isolates unbiasedly recovered from patient specimens in a tertiary hospital in the central part of China. The specimens were collected between January 5, 2013, and July 24, 2018.

### Ethical statement.

This study was approved by the ethics committees under tracking numbers 201806861 and BGI‐IRB 18061.

### Phenotypic tests.

Matrix-assisted laser desorption/ionization-time of flight (MALDI-TOF) mass spectrometry (MS) (Bruker Daltonics, Bremen, Germany) was used to identify the bacteria species. In cases where multiple isolates were obtained from one patient, only the first was kept for the epidemiology analysis. The MIC values were measured by the Vitek-2 compact system (bioMérieux, Marcy l’Etoile, France). The results were interpreted according to the Clinical and Laboratory Standards Institute (CLSI) guideline (27).

### Genomic epidemiological analysis.

WGS was performed by BGISEQ-500 (MGI, Shenzhen, China) using paired-end libraries prepared by MGIEasy Universal DNA Library Prep Set (MGI, Shenzhen, China). The resultant reads were trimmed and qualified by fastp version 0.14.0 (28). SPAdes version 3.10.0 was used for the *de novo* assemblies with *k*-mer sizes of 55, 77, and 99 (29). The assemblies were filtered by several steps to remove genomes representing mixed infections, or contaminated cultures (Table S1 to S3). Only 2,193 unique high-quality assembled K. pneumoniae genomes were retained for the following genomic epidemiology analysis (Table S3). The detailed quality control report is available in Fig. S1. Single-nucleotide polymorphisms (SNPs) were identified by high-quality reads mapping to strain HS11286 (CP003200.1) and the ST25 strain SMU18037509 (CP045661.1). The ST25 genomes (Table S4) were downloaded from the public databases of the National Center for Biotechnology Information (NCBI) and the Pasteur K. pneumoniae MLST database (https://bigsdb.pasteur.fr/klebsiella/, accessed on March 31, 2021). Recombination sites were identified by Gubbins version 2.3.1 (30). The core genome SNPs were identified by “snippy-core” and were filtered by a minimal read depth of 10× and a minimal variant allele frequency of 90% using snippy version 3.2 (https://github.com/tseemann/snippy). The patristic distance of 0.4 given by RAMI version 1.1 defined the clonal groups (31). Each clonal group contains more than 10 unique isolates. The most prevalent sequence type (ST) was used to enumerate each CG (clonal group). The K loci were determined by Kaptive version 0.7.3 (32). Antibiotic resistance genes and plasmid replicon types were identified by ResFinder version 4.0 and PlasmidFinder version 2.1, respectively, with higher than 80% identities and coverages via the ABRicate pipeline (https://github.com/tseemann/abricate) (18, 33).

### Transmission event determination.

The clinical histories (including the admission-discharge dates, the sampling time, the hospitalization wards, treatments, and diagnosis) of the patients were reviewed. The ward information was first used to analyze the transmission chains, from one isolate to its “genetically nearest neighbor (gNN)” forward-clustering results to define “intraward transmission,” “interward transmission” (“wards with imported cases” or “wards with exported cases”), or “transmission hub wards”. The events were epidemiologically defined as happening within the same ward for fewer than 180 days. The matrix of pairwise cgSNPs was obtained by snp-dists (https://github.com/tseemann/snp-dists). Clones with more than 20 unique isolates were selected. A good F1 score (>0.75) was used as the threshold to differentiate the genomically linked and unlinked distances. BEAST version 1.10.4 was used to construct the maximum clade credibility (MCC) tree and calculate the time to the most recent common ancestor (tMRCA) for the transmission subclones (34). Markov chain Monte Carlo (MCMC) procedure was run for 10 million iterations with 10% burn-in. We tried the relaxed clock and the strict clock with the exponential and constant models. The best-supported setting was GTR+estimated+G4, under the exponential model. To validate the transmission events, plausible transmission chains were constructed by SCOTTI (structured coalescent transmission tree inference) (35). SCOTTI simulated the small outbreaks with the consideration of the existence of unobserved and not sampled intermediate hosts. The index case, the root case, the super spreader, and the transmission directions were pinpointed accordingly.

### Statistical analyses.

Statistical analyses were performed using R version 3.6.1. The differences in the prevalence between KpI, KpII-A, KpII-B, and KpIII were tested by the Kruskal-Wallis test. Fisher's exact test was performed for the pairwise differences within each group. *P* values were adjusted for multiple pairwise comparisons using the Bonferroni correction. The distribution of categorical variables were compared across the phenotypically CRKp (carbapenem-resistant Klebsiella pneumoniae) and non-CRKp using Pearson's Chi-squared test. All analysis was two-sided. Heatmap was made using the pheatmap package. Principal-component analysis (PCA) was done using the function of vegdist with the default Euclidean distance and plotted by ggplot2.

### Data availability.

The data that support the findings of this study have been deposited into the China National GenBank (CNGB) Sequence Archive (CNSA) of China National GenBank DataBase (CNGBdb) with accession number CNP0001198 (25, 26).
