# Supplementary material for: Large-Scale Genomic Epidemiology of Klebsiella pneumoniae Identified Clone Divergence with Hypervirulent Plus Antimicrobial-Resistant Characteristics Causing Within-Ward Strain Transmissions
Source: Microbiol Spectr. 2022 Apr 13;10(2):e02698-21. doi: 10.1128/spectrum.02698-21 (PMC9045374; doi:10.1128/spectrum.02698-21)
Supplement: SUPPLEMENTAL FILE 3 — Tables S3 and 7; Fig. S1-5; supplemental text. Download spectrum.02698-21-s003.pdf, PDF file, 0.8 MB [file spectrum.02698-21-s003.pdf]

Supplementary appendix 3

Supplementary tables and figures

Table S1: Detailed assembly results of the 3,061 *Klebsiella pneumoniae* genomes

appendix1.xlsx

Table S2: Identification of best-matching taxa of 3,061 *Klebsiella pneumoniae* genomes

appendix2.xlsx

Table S3: Summary of the filtrations of 3,061 *Klebsiella pneumoniae* genomes.

**Filtration criteria**	**Number (%) of qualified genomes**
Mean depth > 20 ×	3,061 (100%)
K. pneumoniae species by Metaphylan[1]	2,863 (94%)
K. pneumoniae species by mOTU[2]	2,967 (97%)
Number of > 1 Kb contigs < 1,000	3,048 (99%)
Total length of the assembly: 5 – 6.5 Mb	2,969 (97%)
GC content: 40% - 60%	3,027 (99%)
Unique isolate per patient	2,371 (78%)
Overall fulfilled	2,193 (72%)

Table S4: The 62 public *Klebsiella pneumoniae* ST25 genomes downloaded for this study

appendix4.xlsx

Table S5: Clinical records of the 2,193 hosts

appendix5.xlsx

Table S6: Genetic characteristics of the 2,193 *Klebsiella pneumoniae* genomes.

appendix6.xlsx

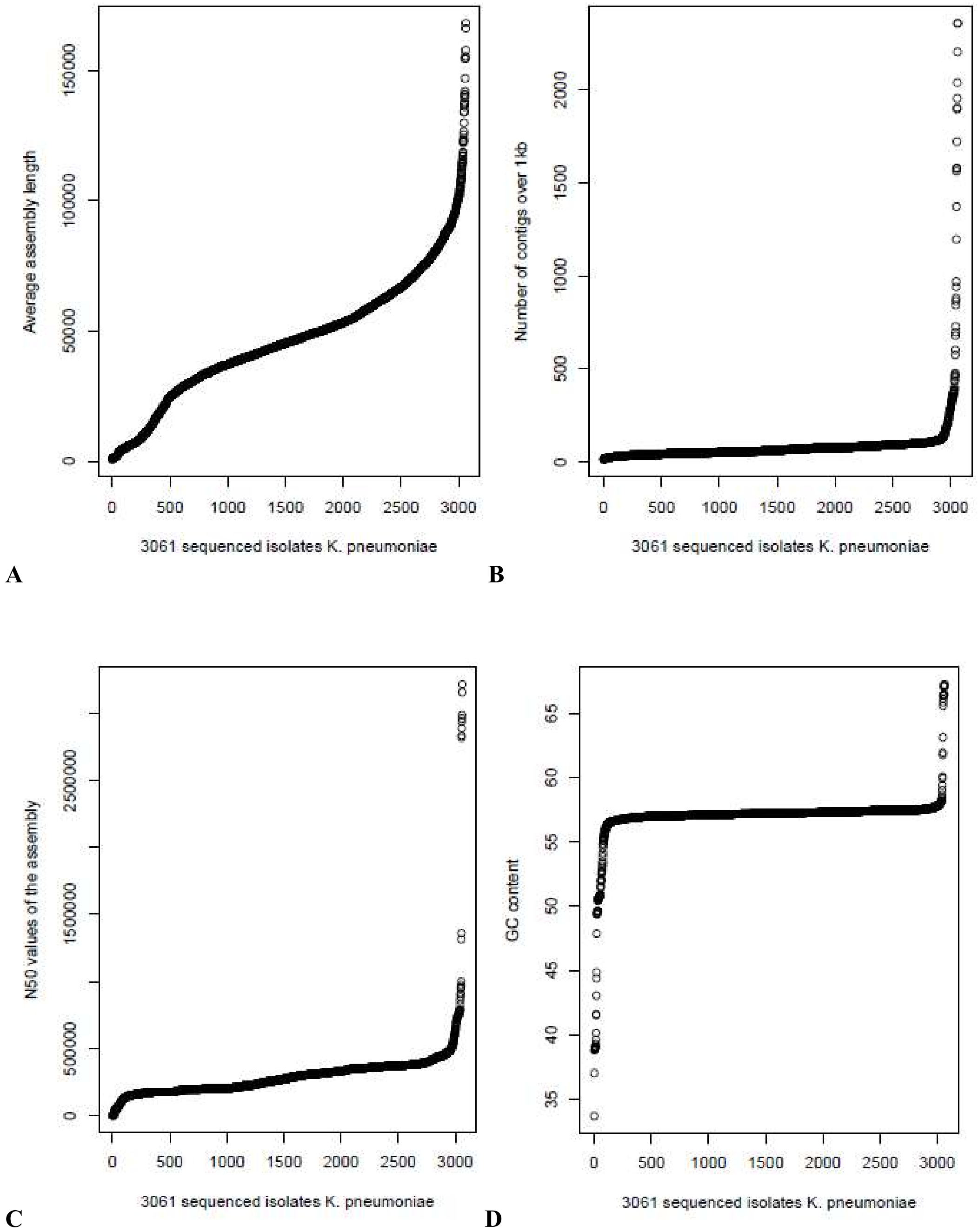

Figure S1: Quality assessment of 3,061 *K. pneumoniae* genomes

(A) Plot of the assembly length. (B) Plot of the number of contigs over 1 Kb. (C) Plot of the N50 values of the assembly. (D) Plot of the GC content.

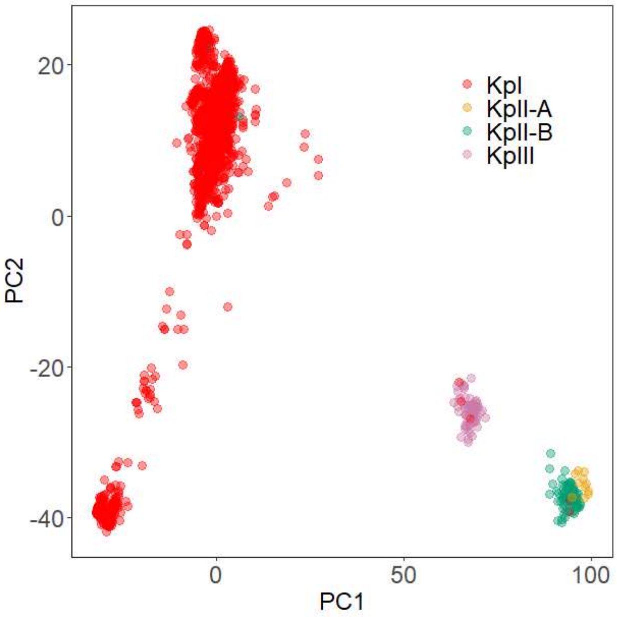

Figure S2: Principal components analysis (PCA) based on the accessory genes of 2,193 *K. pneumoniae* genomes.

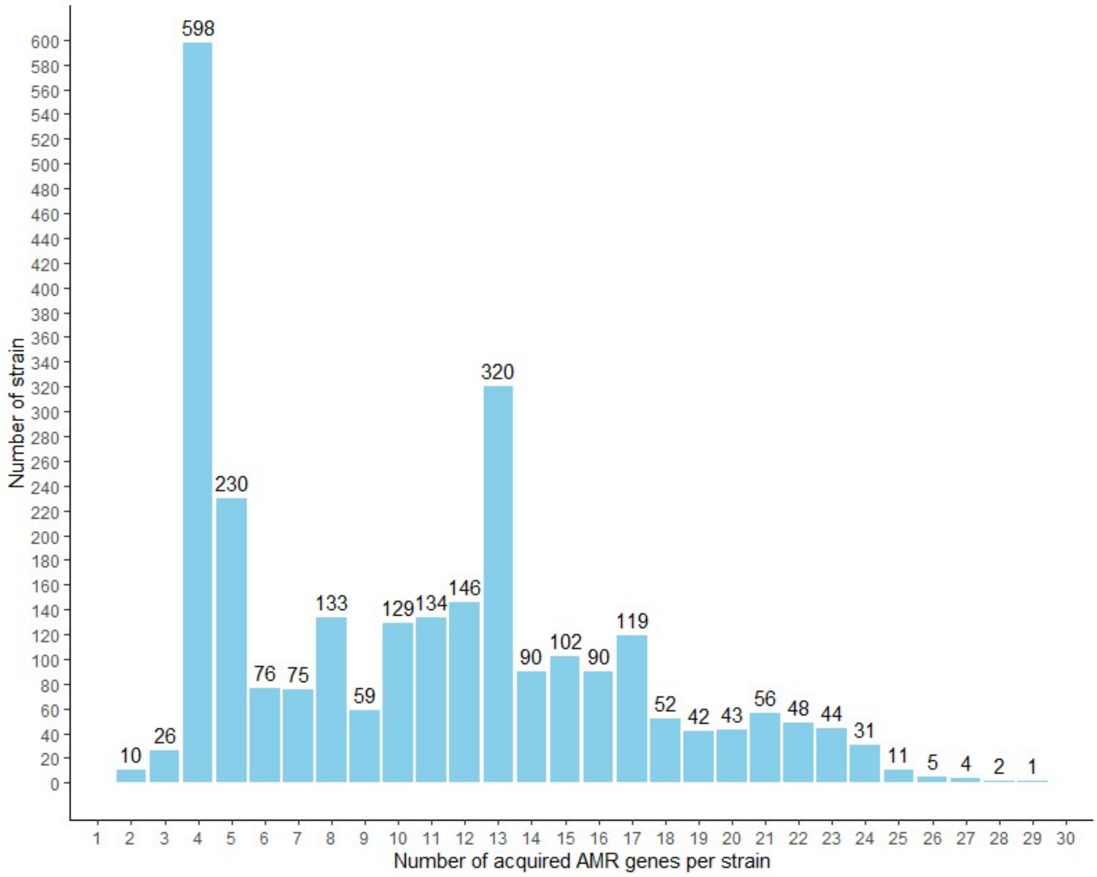

Figure S3: Numbers of acquired antimicrobial-resistant genes in each *K. pneumoniae* genome

Tables S7: Comparison of genetic characteristics of phenotypically carbapenem-resistant *K. pneumoniae* (CRKp) and non-CRKp

	CRKp	non-CRKp	total	*P* value
K. pneumoniae	472	1563	2,035	
CG11	367	39	406	< 0.001
Other CGs	105	1,524	1,629	
**carbapenemase genes**				
$\mathit{bla}_{\text{KPC}}$	84% (397)			
$\mathit{bla}_{\text{NDM}}$	6% (30)			
$\mathit{bla}_{\text{IMP}}$	3% (12)			
**extended spectrum β-lactamase (ESBL) genes**				
$\mathit{bla}_{\text{CTX-M-3}}$	37	183	220	< 0.001
$\mathit{bla}_{\text{CTX-M-14}}$	8	177	185	< 0.001
$\mathit{bla}_{\text{CTX-M-15}}$	34	153	187	< 0.001
$\mathit{bla}_{\text{CTX-M-65}}$	292	22	314	< 0.001
$\mathit{bla}_{\text{CTX-M-55}}$	6	26	32	< 0.001
Other $\mathit{bla}_{\text{CTX-M}}$	6	49	55	< 0.001
$\mathit{bla}_{\text{OXA}}$	23	106	129	< 0.001
$\mathit{bla}_{\text{SHV}}$	472	1557	2029	< 0.001
$\mathit{bla}_{\text{TEM}}$	378	453	831	0.01
$\mathit{bla}_{\text{SFO}}$	10	16	26	0.24
**other *bla* genes**				
$\mathit{bla}_{\text{DHA}}$	24	96	120	< 0.001
$\mathit{bla}_{\text{LAP}}$	13	186	199	< 0.001
$\mathit{bla}_{\text{CMY}}$	6	2	8	0.16

Table S8: Demographic characteristics and clinical outcomes of the patients with *K. pneumoniae* CG25 infections.

appendix7.xlsx

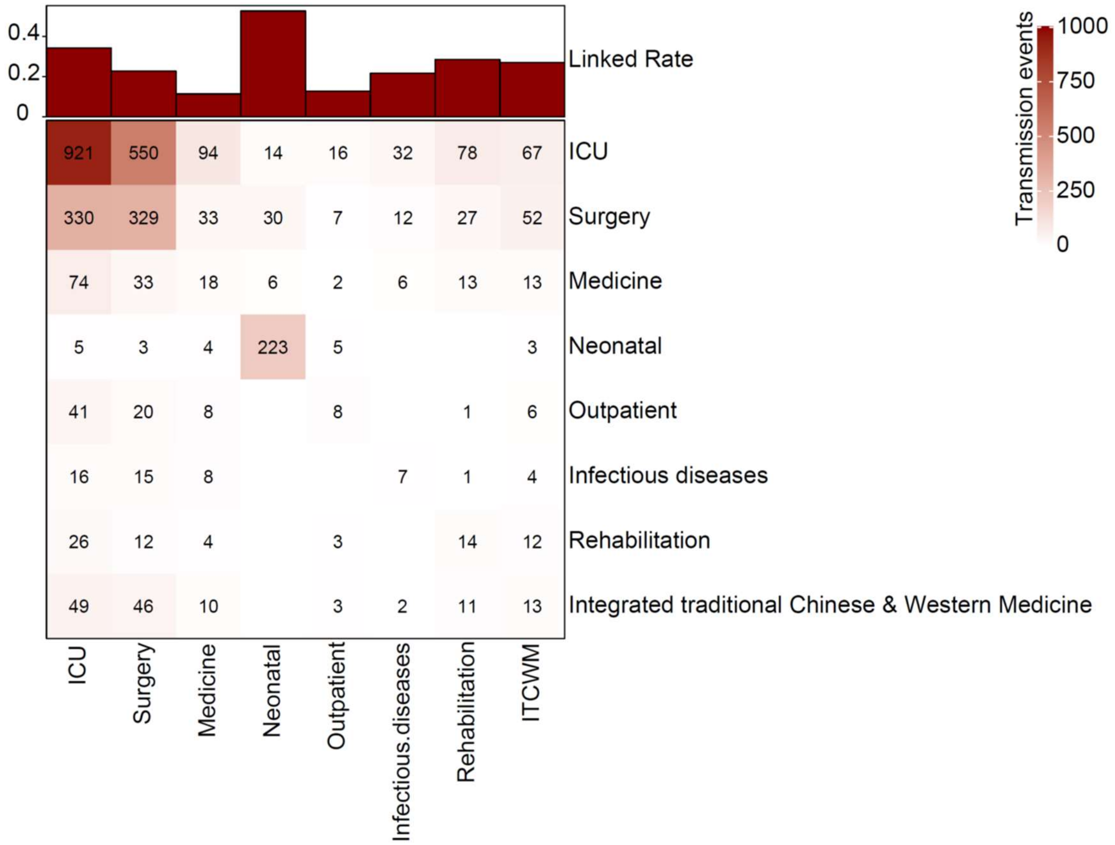

Figure S4: The distribution of possible strain transmission events

The linked rates were calculated by the linked transmissions and the total infection cases in each ward.

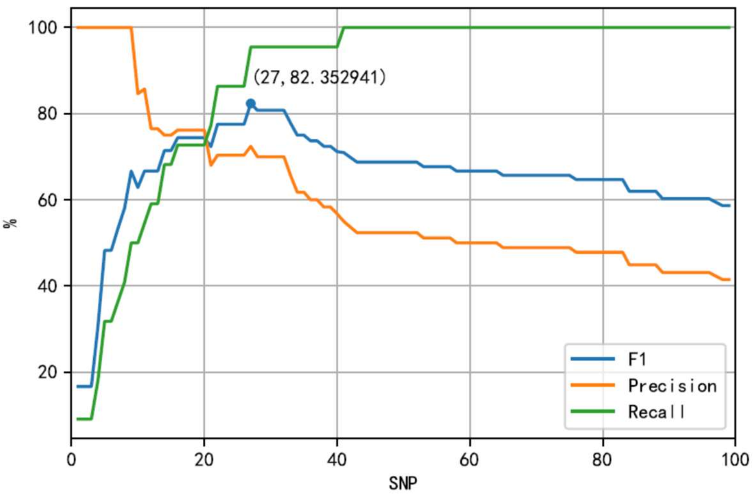

A

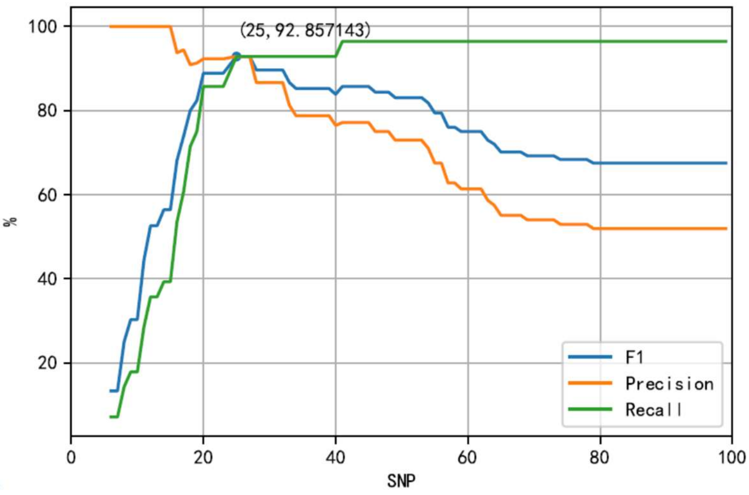

B

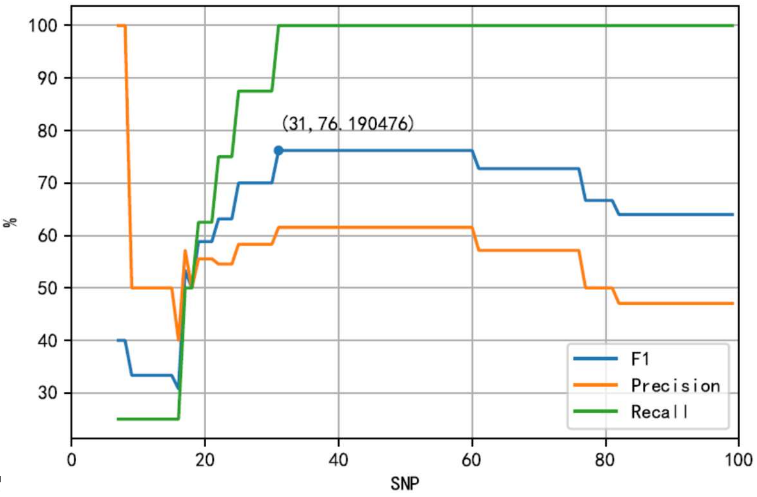

C

Figure S5: F1 scores of the pairwise core-genome single nucleotide polymorphisms (cgSNPs) in (A) ST25, (B) ST307, and (C) ST20

The pairwise cgSNP cutoff and the F1 score was in the bracket.

List of symbols and abbreviations

ST	sequence type
WGS	whole-genome sequencing
SNP	single-nucleotide polymorphism
cg	core-genome
KpI	*K. pneumoniae*
KpII-A	*K. quasipneumoniae subsp. quasipneumoniae*
KpII-B	*K. quasipneumoniae subsp. similipneumoniae*
KpIII	*K. variicola*
KPC	*Klebsiella pneumoniae* carbapenemase
CG	clonal group
KL	K loci
hv	hypervirulent
AMR	antimicrobial-resistant
ICU	intensive care units
MDR	multidrug-resistant
ARG	antimicrobial-resistant gene
CR	carbapenem-resistant
MALDI-TOF MS	Matrix-Assisted Laser Desorption/Ionization-Time of Flight Mass Spectrometry
MIC	minimum inhibitory concentration
CLSI	Clinical and Laboratory Standards Institute
LB	Luria-Bertan
bp	base pair
Kb	kilo base
Mb	mega base
Gb	giga base
ML	maximum-likelihood
GTR	general time-reversible
MLST	multi-locus sequence typing
MCC	maximum clade credibility
tMRCA	time to the most recent common ancestor
CRKp	carbapenem-resistant *Klebsiella pneumoniae*
ESBL	extended spectrum beta-lactamase
MLSB	macrolide-lincosamide-streptogramin B
HPD	highest posterior density
Inc	incompatibility
HGT	horizontal gene transfer
ICU	intensive care unit
VLBWI	very low birth weight infant
etc.	*et cetera* / and so on

1. Truong DT, Franzosa EA, Tickle TL, et al. MetaPhlAn2 for enhanced metagenomic taxonomic profiling. *Nat Methods* 2015; **12**(10): 902-3.
2. Milanese A, Mende DR, Paoli L, et al. Microbial abundance, activity and population genomic profiling with mOTUs2. *Nat Commun* 2019; **10**(1): 1014.
